# Dysphotopsias or Unwanted Visual Phenomena after Cataract Surgery

**DOI:** 10.3390/life13010053

**Published:** 2022-12-24

**Authors:** Ambroz Pusnik, Goran Petrovski, Xhevat Lumi

**Affiliations:** 1 Eye Hospital, University Medical Centre Ljubljana, 1000 Ljubljana, Slovenia; 2 Center for Eye Research and Innovative Diagnostics, Department of Ophthalmology, Oslo University Hospital, 0450 Oslo, Norway; 3 Institute of Clinical Medicine, Faculty of Medicine, University of Oslo, Kirkeveien 166, 0450 Oslo, Norway; 4 Department of Ophthalmology, University of Split School of Medicine and University Hospital Centre, 21000 Split, Croatia; 5 Faculty of Medicine, University of Ljubljana, 1000 Ljubljana, Slovenia

**Keywords:** cataract surgery, unwanted visual phenomena, positive dysphotopsia, negative dysphotopsia, intraocular lens

## Abstract

Dysphotopsias are unwanted visual phenomena that occur after cataract surgery. They represent some of the most common reasons for patient dissatisfaction after uncomplicated surgery for cataract phacoemulsification with in-the-bag intraocular lens (IOL) implantation. Depending on the form of the optical phenomenon and the effect it poses on vision, dysphotopsias are divided into positive and negative type. Positive dysphotopsias are usually described by patients as glare, light streaks, starbursts, light arcs, rings, haloes, or flashes of light. Negative dysphotopsias are manifested as an arc-shaped shadow or line usually located in the temporal part of the visual field, similar to a temporal scotoma. In addition to their different clinical manifestations, positive and negative dysphotopsia also have different risk factors. Even though up to 67% of patients may experience positive dysphotopsia immediately after surgery, only 2.2% of the cases have persistent symptoms up to a year postoperatively. Surgical intervention may be indicated in 0.07% of cases. The incidence of negative dysphotopsias is up to 26% of all patients; however, by one year postoperatively, the symptoms usually persist in 0.13 to 3% of patients. For both types of dysphotopsia, preoperative patients’ education, accurate preoperative diagnostics, and use of an appropriate IOL design and material is mandatory. Despite all these measures, dysphotopsias may occur, and when noninvasive measures fail to improve symptoms, a surgical approach may be considered.

## 1. Introduction

Dysphotopsias are undesirable optical phenomena caused by external light source superimposing unwanted patterns over the true retinal image [1,2,3]. The term dysphotopsia is usually mentioned as a consequence of cataract surgery with implantation of an intraocular lens (IOL), although they can less commonly occur in phakic patients as well [2,4]. Two main types of dysphotopsia have been described: positive dysphotopsia (PD) and negative dysphotopsia (ND) [1,5]. Patients with PD often describe unwanted visual phenomena such as glare, haloes, appearance of light streaks, and light arcs or flashes that are caused by external light sources (e.g., lamps and car lights) [6]. Patients with ND typically experience a temporal dark crescent-shaped shadow [7]. Despite the fact that these conditions present differently and have different causes, they can also occur simultaneously [8]. Dysphotopsias are of transient nature in most of the pseudophakic patients. Therapeutic measures are needed in cases of long-term persisting problems [5].

## 2. Positive Dysphotopsia

PD after cataract surgery is described by patients as glare (due to high refractive index (RI) and reflectance of the IOL), light streaks and starbursts (due to backscatter from the IOL and microsaccades, exacerbated by higher RI of the lens), light arcs (seeing the edge of the IOL, usually at night), rings and haloes (more commonly seen with multifocal IOLs (MFIOL)), or flashes of light (reflections of peripheral light rays off the edge of the IOL) [6]. Patients usually experience these phenomena near the visual axis, especially in low mesopic or scotopic conditions when the pupils dilate [2,6,9]. PD is evoked by an external light source coming obliquely from the periphery [3,8,9]. 

In the early period after cataract surgery, PD is experienced by up to 67% of patients [5,10]. In most cases, symptoms spontaneously resolve; however, in up to 2.2% of patients, symptoms persist up to one year after surgery [11]. According to Davison JA, in 0.07% of cases, an additional procedure is required to resolve PD [6]. The most commonly mentioned factors for occurrence of PD are IOL shape [12], sharp-edged design [11], RI [8], pupil size [2], and IOL size [10,13]. PD needs to be distinguished from single light streak optical phenomena caused by posterior lens capsule striae after IOL implantation (i.e., Maddox rod effect) which demand a different line of treatment [3]. PD also needs to be distinguished from entoptic phenomena and photopsias, which are not caused by external light sources such as vitreomacular traction [3,8,9,14].

### 2.1. IOL Shape

A clinical study on 289 patients showed that ovoid IOL shape significantly contributes to the PD occurrence [12]. These findings were supported by laboratory scatterometry investigations [12]. Based on these findings, the use of oval-shaped IOLs has decreased [2,15,16]. The anterior and posterior IOL surface curvature also seems to be an important factor for PD development [17,18]. IOLs with anterior radius curvature of ≤17 mm would minimize surface reflections [18]. A combination of an unequal biconvex IOL design with a flatter anterior surface curvature and high IOL-refraction index increases the internal light reflections and causes more intensive and focused illumination of the retina which can result in unwanted glare images [17,18]. PD can thus be avoided by using equi-biconvex IOLs which allow the inner-reflected rays to fall onto the retina in a more dispersed fashion, causing less intense retinal illumination [17]. 

If the incidence angle of the light rays onto the IOL exceeds a critical angle of about 35° off the visual axis, it can create an internal reflection in the IOL which projects onto the temporal retina [15,17]. The critical angle of the acrylic IOL is smaller than that of silicon IOLs, which can cause an increased internal reflection with the former; the intensity of these reflections can be over 1000 times higher than those from an unaccommodated human lens [15]. Use of acrylic IOLs has, otherwise, increased due to their plausible foldability, which is important for their implantation. Interestingly, previously used PMMA lenses caused little to no dysphotopsias [1,19,20].

### 2.2. Sharp-Edge Design

**Figure 1:**
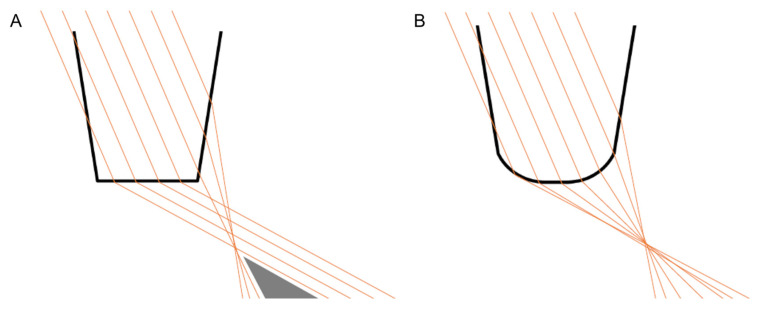
IOL shape: sharp-edge (**A**) versus rounded-edge (**B**) design and the aberrant visual phenomena associated with incident light coming from the temporal direction.

A sharp-edge design of IOL optic was recognized as a contributing factor for PD development (Figure 1A) [11,12,15]. In a ray-tracing analysis, Holladay et al. showed that a sharp-edge IOL design increases the probability of a thin, ring-like image projecting onto the midperipheral retina [15]. The same study showed reduced probability of PD by dispersing the reflected rays with rounded-edge IOL design (Figure 1B) [15]. However, sharp-edged IOLs are still commonly in use, as they slow down posterior capsule fibrosis [21]. Meacock et al. suggested using square-edge IOL with a textured edge to prevent unwanted glare symptoms [22]. However, a study by Franchini et al. did not show any significant decrease in PD incidence by using square-edge IOLs with frosted edge [23]. Moreover, the use of such IOLs could result in decreased contrast sensitivity [23]. 

### 2.3. IOL Materials and Refraction Index

The effect of the material of IOL and RI on the occurrence of PD symptoms is not unequivocally explained. A 2020 study found that hydrophobic IOLs with a higher RI, especially acrylic IOLs, increase the incidence of PD [8]. RI of the aqueous is 1.336, PMMA IOL is 1.49, acrylic hydrophilic IOL is 1.43, acrylic hydrophobic IOL is 1.44–1.55, and silicon IOL is 1.46 [24]. The critical angles are acrylic IOLs 59.5°, PMMA IOLs 63.7°, and silicon IOLs 66.2° [24]. This makes the acrylic IOLs most likely to undergo internal reflection [24], which is supported by the theoretical explanation that a higher RI enhances light reflection within the IOL and increases the probability for PD [6,8,17]. However, a retrospective comparison study of IOLs with different RI by Radmall et al. could not confirm these predictions [25]. Another study on 600 patients that compared four different types of IOLs could not find a connection between IOL material and occurrence of PD either [13]. 

### 2.4. Pupil and IOL Size

**Figure 2:**
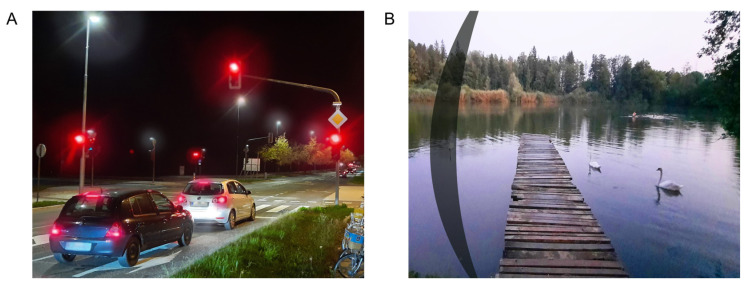
Visual phenomena in positive dysphotopsia—glare (**A**), and negative dysphotopsia—temporal arc-shaped shadow (**B**).

Pupil size is commonly mentioned as a possible factor for PD development. In theory, a larger pupil would expose the IOL’s edge to more light rays, which by internal reflection would cause an illuminated arc-like pattern on the peripheral retina [11,15]. This theoretical explanation is supported by patients complaining of glare symptoms occurring in low mesopic or scotopic light conditions such as reading at night with a light source on the side or driving at night (Figure 2A) [2]. It has been suggested that in such cases, miosis may be beneficial to control glare symptoms [11]. However, clinical findings about pupil size correlating to PD seem contradictory. A review article from 2021 recommends pharmacological miosis as a conservative management method in highly symptomatic patients [3]. On the other hand, Davison JA states that the use of miotic therapy does not improve PD symptoms [6]. 

PD occurrence may also depend on the IOL diameter. Bournas et al. found that a smaller IOL optic diameter is associated with higher odds of optic phenomena. Specifically, 5.5 mm diameter IOLs were linked to an increased risk for dysphotopsia compared to 6 mm diameter IOLs [13]. Similar findings were described by Bonsemeyer et al. that found 7 mm diameter IOLs to reduce both PD and ND incidence compared to 6 mm diameter IOLs [10].

## 3. Negative Dysphotopsia

In ND, an arc-shaped shadow or line is usually located in the temporal visual field similar to a temporal scotoma (Figure 2B) [3,6,13]. The scotoma may be evaluated by visual field testing [26]. ND is evoked by an external light source that is typically temporally oriented [8,27]. Patients most commonly experience this phenomenon in photopic conditions when the pupil is narrow [27,28]. ND is a diagnosis of exclusion where other possible ocular and neuro-ophthalmological pathologies should be excluded [29]. The incidence of ND is highest on the first week after cataract surgery; it is noted by up to 26% of all patients [30]. However, by one year after surgery, the symptoms usually persist in 0.13 to 3.2% of patients [31,32]. Five years after surgery, symptoms may persist in only 1.5% of patients [33]. ND seems to occur more commonly in left eyes or in women [29,31,33]. The etiology of ND is not clearly defined, and the cause seems to be multifactorial. Holladay and Simpson categorized the risk factors for ND development into three groups: anatomic characteristics (pupil size, hyperopia, and angle kappa), IOL properties (IOL surface steepness, edge design, dioptric power, and refraction index), and surgical technique for cataract removal (optic–haptic junction orientation and position of nasal anterior capsule to the IOL surface) [34]. There is also a possibility that central nervous system adaptation mechanisms could be involved in the ND development, although they are not yet clearly understood [35,36]. It may be possible that transient and persistent ND have different causes [31,33].

### 3.1. The Illumination Gap Theory

**Figure 3:**
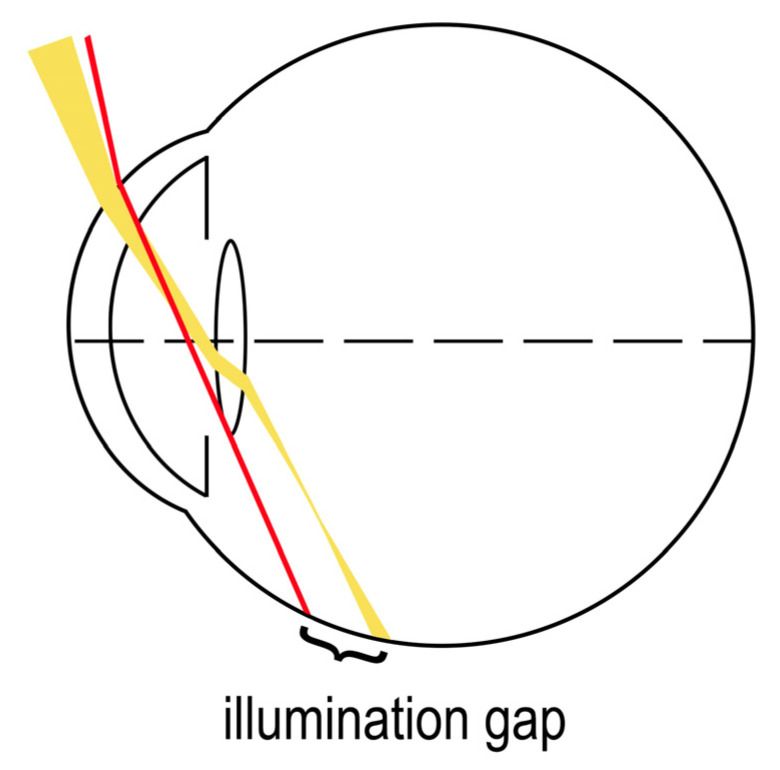
“Illumination gap” theory—a gap between the different refraction of rays hitting the IOL optic periphery (yellow) and the rays that miss the IOL (red).

The most supported working theory for temporal visual field shadow occurrence in pseudophakic patients with ND is the illumination gap of the nasal retina [34]. The illumination gap is caused by different refraction of rays that hit the IOL optic periphery to those that miss the IOL (Figure 3) [3,7,34]. The illumination gap is bounded posteriorly by the rays refracting on IOL optic periphery and anteriorly by the rays missing the IOL which are not refracted [7,34,37]. The location of the illumination gap usually corelates well with the ND symptoms described by patients [3,7,34].

### 3.2. Visual Field Defects

A study of eleven patients with ND showed that symptoms may be objectively evaluated by kinetic perimetry testing as statistically significant constrictions of the peripheral temporal and inferior visual field [26]. Similar findings were observed by Masket et al. [35]. Since kinetic perimetry measures the extension of the visual field up to 90 degrees and the visual field of a normal individual can extend up to 110 degrees temporally, it is possible that scotomas reaching to the extreme periphery of the temporal visual field are being underestimated [26]. Masket et al. found that translucent or opaque occlusion of the fellow eye resulted in subjective improvement of symptoms [35]. Furthermore, a pilot kinetic perimetry investigation on four ND patients found inferotemporal peripheral scotoma to be larger in extent with both eyes fully opened compared to a peripherally occluding contact lens being applied to the contralateral eye [36]. These findings raise the possibility of a neuroadaptive component to the ND [35]. 

### 3.3. Patient Anatomy

A study by Osher RH found that permanent ND symptoms could be a result of interaction between the IOL edge and anatomical predisposition of patients [31]. One of the fundamental predispositions for an illumination gap to be perceived as a temporal arching shadow is the presence of functional nasal retina, which extends more anteriorly compared to the temporal retina [7,31,38]. Other anatomical factors present together include prominent eyeball, shallow orbit, smaller and decentered pupil, hyperopia and large angle kappa, and large angle alpha, which can also increase the incidence of ND [31,34,38,39]. A ray-tracing analysis by Holladay et al. showed that the distance between IOL and iris, ranging from 0.06 to 1.23 mm for acrylic and 0.06 to 0.62 mm for silicon IOLs, may be a factor for ND development [7]. However, the claim that a larger distance between iris and IOL increases the rate of ND has not been confirmed by later studies [27,32,40].

A ray tracing analysis study on computer eye models by Holladay and Simpson showed that a smaller photopic pupil is a significant factor for temporal shadow occurrence [34]. The retinal shadow occurred in pseudophakic conditions with a small 2.5 mm pupil diameter, while the shadow disappeared when the pupil was 5 mm wide [34]. This was attributed mostly to larger ray dispersion in wider pupils which reduces the chance for an illumination gap to occur [7,34]. A 2011 study by Masket and Fram noted an increase in ND symptoms with miotic agents and their improvement after the application of mydriatic agents [27]. Additionally, there are case reports of patients complaining of more intensive ND symptoms in bright photopic conditions [28]. Nasal location of the pupil relative to the eye’s optical axis (>2.6° or 0.3 mm on the cornea) can be the cause of exposure of the nasal retina to light rays [7,24].

Another study on 95 patients showed that hyperopic subjects might be more susceptible for ND development [30]. Large angle kappa—the angle between the visual axis (an imaginary line connecting the point of gaze fixation and the fovea) and the pupil axis (an imaginary line running through the pupil center perpendicular to the cornea)—might also contribute to ND development [34,41]. Angle kappa is larger in hyperopic patients as significant correlation exists between angle kappa values and positive refractive errors [42]. Furthermore, patients’ angle alpha—the angle between the visual axis and the optical center of the cornea—could be another factor for ND development [7,24]. A large angle alpha causes the eye to be turned more temporally and thus increases the exposure of functional nasal retina [7,24]. A complex interplay of all the factors mentioned above seems to increase ND incidence.

### 3.4. IOL Properties

ND can occur with different types of IOLs, irrespective of shape and material [3,27,28]. Hydrophobic, hydrophilic, acrylic, and silicon IOLs can all be associated with ND [28,29,32]. However, ND could be more commonly associated with acrylic IOLs with a sharp-edge design and less commonly with silicone IOLs with a rounded-edge design [2,34,43]. According to Holladay et al., the silicone IOLs reduce the width of the illumination gap and moves it more anteriorly [7]. It is thus less likely for the illumination gap to form on the functional retina [7]. Edge shape is, therefore, an important factor, since rounded edges disperse the rays and thus reduce or eliminate the illumination gap [7]. An irregular IOL edge could also cause sufficient ray scatter to eliminate the gap [44]. The effect of the IOL shape on ND development has been further highlighted in a computer ray-tracing analysis by Holladay and Simpson [34]. The same research mentioned a number of IOL properties that could affect ND incidence, including high RI, higher dioptric power, equi-biconvex or plano-convex shape, negative aspheric surface, and IOL diameter [34]. High RI of the optic material, in particular, acrylic IOLs, moves forward the anterior and the posterior border of the shadow reducing its width compared to silicone IOLs. [7,24]. Modification of the IOL design and diameter could reduce ND [10,45,46]. A concave region on the peripheral posterior surface of a biconvex IOL may prevent ND by increasing the area of illuminated peripheral retina and narrowing the illumination gap [45]. A ray-tracing analysis suggested that 7.0 mm optic diameter IOLs enlarge and shift the illumination gap more peripherally compared to 6.0 mm diameter IOLs [46]. This effect was, however, more pronounced with a lower RI IOL [46]. It could be less likely for the shifted illumination gap to fall on the functional retina and be perceived as troublesome [46]. A study on 86 patients comparing two hydrophilic acrylic IOLs with the same RI showed the 7.0 mm optic diameter IOL to have reduced ND incidence compared to the 6.0 mm diameter IOL [10].

### 3.5. Surgical Techniques

ND occurrence has been reported after IOL implantation in the capsular bag but not after ciliary sulcus or anterior chamber implantations [27]. One study suggests that a nasal anterior capsule overlying the anterior nasal part of the IOL optic could be a factor determining the presence of ND by reducing the intensity of rays transmitted to the retina due to ray reflections [34]. ND could, thus, be alleviated if IOL optic covered the anterior capsulotomy edge [27]. A surgical technique of reverse optic capture was developed, where the edge of the IOL optic is secondarily elevated above the anterior capsulorhexis while leaving the IOL haptics in the capsular bag [27,29]. A study by Masket et al. showed this technique to be highly successful in eliminating or preventing ND [29]. However, the intervention can be linked to postoperative complications, such as earlier opacification of the posterior lens capsule, capsular block syndrome, iris chafe, and postoperative myopic refractive error (myopic shift) [27,29]. Case reports of successful ND treatment by laser capsulotomy of the nasal anterior capsule further suggests that ND is likely caused by the anterior capsulotomy edge with in-the-capsular-bag implantations [47,48]. 

Optic–haptic junction positioning could also affect ND development. A study on 305 patients found a 2.3-fold decrease in ND incidence one day after cataract surgery when one of the two optic–haptic junctions of the IOL was positioned inferotemporally compared to the control group with vertical positioning of the junctions [43]. However, one month after surgery, the difference in ND incidence was no longer statistically significant [43]. A study by Holladay et al. also found horizontal haptic positioning to reduce ND incidence [7]. Another study by Manasseh et al. found ND incidence 4 weeks after surgery to be decreased from 16% to 8% when optic–haptic junctions were horizontally oriented [49]. A ray-tracing analysis by Erie et al. suggested that light rays missing the IOL optic but hitting the optic–haptic junction are completely internally reflected, thus not forming the anterior boundary of the illumination gap on the peripheral retina [37].

Osher RH proposed temporal corneal incision causing localized corneal edema to explain transient, but not persistent, ND symptoms [31]. Osher RH observed a crescent-shaped shadow near the pupil when light was passing through the incision from a temporal angle [31]. The disappearance of ND symptoms weeks after surgery could be associated with resolution of corneal edema [31]. Similarly, a 5-year follow-up study on 320 patients showed hydration of the temporal corneal wound at the end of surgery to possibly increase the risk for transient ND [33]. In this study, 13% of the patients who received wound hydration experienced ND compared to 5% who did not receive wound hydration [33]. 

## 4. Dysphotopsia with Multifocal and Toric IOLs

MFIOLs are associated with higher incidence of PD symptoms compared to monofocal IOLs [50,51,52]. Pieh et al. showed that diffractive MFIOLs are more commonly associated with glare phenomena compared to refractive MFIOLs, although the difference might not be clinically relevant [53]. As the depth of the field increases, so do dysphotopsias, and then visual quality decreases. The reason for this, especially with diffractive technology, is how the depth of the field and dysphotopsias are related to each other. MFIOLs can partially satisfy patients’ expectations, but they also produce gaps in the range of vision and are associated with halos, glare, and reduced contrast sensitivity [54]. The symptoms could be induced by out-of-focus images produced by the MFIOLs [55]. De Vries et al. showed that glare occurs in 38% of eyes after MFIOL implantation [56]. Mendicute et al. noted PD symptoms in 80% of patients after MFIOL implantation, although only 5% of the patients found the symptoms as bothersome [57]. A study comparing three different types of MFIOL found that six months after MFIOL implantation, 65% to 79% of patients reported haloes and 43% to 64% reported glare symptoms [58]. Starbursts were one of the least commonly reported PD symptoms [58]. The same study also suggested that a higher number of diffractive rings may cause more intense symptoms and MFIOLs with a lower number of diffractive rings may provide patients with better quality of vision [58]. After toric IOL implantation, 15–30% of patients complain of moderate or severe PD symptoms [59,60]. Three months after surgery, severe symptoms persist in 7.5% of patients [60]. In the last years, extended depth-of-focus IOLs (EDOF) have been introduced as presbyopia-correcting IOLs with the possibility of reducing PD occurrence [61,62]. However, a 2021 meta-analysis study comparing EDOF and MFIOL implantation outcomes could not find significant advantages of EDOFs compared to MFIOLs [63]. Monovision surgical techniques for correcting presbyopia may induce less PD compared to MFIOL implantation [64]. On average, only 70% of patients who receive a presbyopia-correcting IOL are happy with their level of visual quality, and only 66% are happy with their level of dysphotopsia at 1 month postoperatively [65].

## 5. Preventive and Treatment Measures for Dysphotopsias

**Figure 4:**
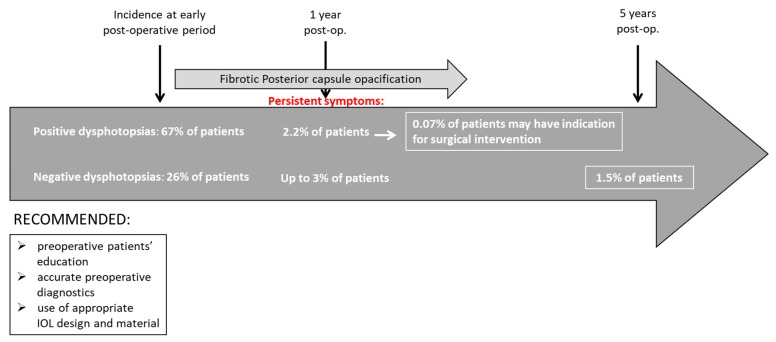
Time course of dysphotopsia symptom persistence.

Both, PD and ND can occur separately or as a combination of both [11]. Postoperatively, the spontaneous opacification of the nasal capsule leads to spontaneous resolution of ND (diffuser effect) [66,67,68,69], while posterior capsule opacification causes light scatter, thus reducing retinal contrast and threshold sensitivity [70]. Capsular bag contraction can cause anterior axial movement of the IOL, reducing the axial space behind the iris, which could also be the reason for dysphotopsias [7]. The symptoms PD and ND cause are usually transient symptoms, thus the first-line measures alleviating them involve patient education, counseling, and noninvasive approaches [5]. A time course of dysphotopsia symptom persistence is presented in Figure 4.

**Figure 5:**
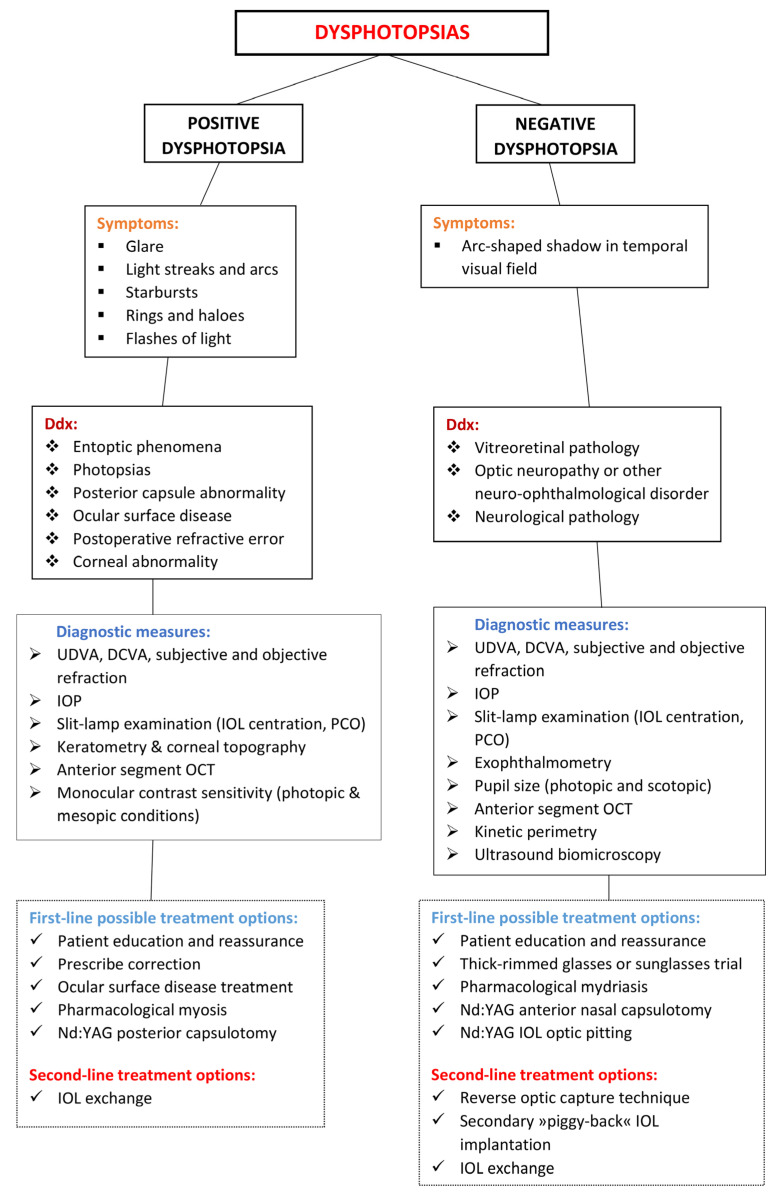
Course of treatment flowchart for patients with dysphotopsia.

PD symptoms can resolve by correcting any postoperative refractive error, treating coexisting ocular surface diseases (e.g., dry eye syndrome), treating posterior capsular opacification, or by inducing pharmacological miosis [8,71,72]. Any corneal abnormalities should be examined, including sequels of previous refractive surgery, presence of epithelial basement membrane disease, or microcystic oedema. Diagnostic measures should include measuring uncorrected distance visual acuity (UDVA), uncorrected near visual acuity (UNVA), distance corrected visual acuity (DCVA), distance corrected near visual acuity (DCNVA), subjective and objective spherical equivalent refraction, monocular contrast sensitivity testing, intraocular pressure (IOP) measurement, and slit-lamp examination [10,57]. Diagnostic imaging should include keratometry, corneal topography, and anterior segment OCT [10]. A flowchart showing the course of treatment of a patient with dysphotopsia is presented in Figure 5.

When noninvasive measures fail to improve symptoms, a surgical approach may be considered. IOL exchange has been reported to be successful (Figure 5) [6]. Usually, the original IOL is replaced with an IOL that is associated with a lower risk of PD occurrence [5]. A study by Masket et al. showed that exchanging the original IOL for a secondary IOL with a lower RI improved PD symptoms in 84% of the patients [8]. The same study also found an IOL exchange for a square-edged 3-piece silicone IOL to be the most successful in treating PD [8].

Adaptation might play a role in long-term decrease of ND symptoms [31,65]. Examination of symptomatic patients should include UDVA, DCVA, subjective refraction, IOP measurement, photopic and scotopic pupil size measurement, exophthalmometry, slit-lamp examination, and anterior segment OCT [31]. Kinetic perimetry may reveal peripheral visual field defects [26,31]. Ultrasound biomicroscopy may be useful to determine IOL-to-iris distance [31]. Thick-rimmed glasses or sunglasses may reduce symptoms by blocking the temporal field of view [31,65]. Symptoms can also be alleviated by pharmacologic mydriasis which increases the illumination of the peripheral retina [27,73]. A limited number of case reports showed that a Nd:YAG laser capsulotomy of the anterior nasal capsule may be effective in some patients with ND [47,48]. Nd:YAG laser capsulotomy of the posterior lens capsule in ND patients is not recommended as ND symptoms are not a result of the opacification of the posterior lens capsule and can make later surgical IOL exchange more difficult [9,31]. A case report by Feng et al. proposed using a Nd:YAG laser to create a cluster of pits in the nasal part of the IOL optic (Figure 5). The laser-induced pits could increase nasal light scatter and resolve ND [74]. However, the pits in the IOL could cause irreversible side effects, such as glare [74,75].

Surgical measures may be considered if troublesome ND symptoms persist for several months or more [29,76]. Reverse optic capture technique, sulcus placement of the IOL, and implantation of a secondary “piggy-back” IOL might improve ND symptoms [27]. A ray-tracing analysis using patients’ biometric parameters showed that implanting an additional sulcus-fixated IOL increases light irradiance of the peripheral retina [77]. This increase was greater in patients with complete resolution of ND symptoms after a supplementary sulcus-fixated IOL implantation was performed compared to those without complete symptom resolution [77]. The reverse optic capture technique ensures that the anterior surface of the IOL is fully exposed to light and is not covered by the anterior capsule edge, and it allows the IOL’s optic to move more anteriorly [34]. By implanting a secondary IOL in the ciliary sulcus, a “piggy-back” IOL, a larger area of the peripheral retina gets illuminated [73], which improves ND in approximately 73% of the cases [73]. An IOL exchange can also alleviate symptoms, although it is not always successful [29,32]. An IOL exchange for a sulcus-fixated round-edged silicon IOL may also be successful (Figure 5) [32,38,76].

## 6. Conclusions

Dysphotopsias are very often the cause of patient dissatisfaction after uncomplicated cataract surgery. In most cases, they are transient in nature and disappear spontaneously within a few weeks or in the first year after surgery. The incidence of these adverse events has been increasing in recent years due to the increasing use of multifocal and toric IOLs in cataract surgery. Due to the frequent occurrence, it is very important that the surgeon informs the patient about the possibility of these phenomena and their harmless nature. In some cases, these phenomena are persistent. Conservative or pharmacological management is possible especially for positive dysphotopsias, but it is often ineffective. In persistent cases with bothersome symptoms, it may be necessary to surgically solve the problem. 

## Data Availability

Not applicable.
